# When the Sun Prickles Your Nose: An EEG Study Identifying Neural Bases of Photic Sneezing

**DOI:** 10.1371/journal.pone.0009208

**Published:** 2010-02-15

**Authors:** Nicolas Langer, Gian Beeli, Lutz Jäncke

**Affiliations:** Psychological Institute, Division of Neuropsychology, University of Zurich, Zurich, Switzerland; Freie Universitaet Berlin, Germany

## Abstract

**Background:**

Exposure to bright light such as sunlight elicits a sneeze or prickling sensation in about one of every four individuals. This study presents the first scientific examination of this phenomenon, called ‘the photic sneeze reflex’.

**Methodology and Principal Findings:**

In the present experiment, ‘photic sneezers’ and controls were exposed to a standard checkerboard stimulus (block 1) and bright flashing lights (block 2) while their EEG (electro-encephalogram) was recorded. Remarkably, we found a generally enhanced excitability of the visual cortex (mainly in the cuneus) to visual stimuli in ‘photic sneezers’ compared with control subjects. In addition, a stronger prickling sensation in the nose of photic sneezers was found to be associated with activation in the insula and stronger activation in the secondary somatosensory cortex.

**Conclusion:**

We propose that the photic sneeze phenomenon might be the consequence of higher sensitivity to visual stimuli in the visual cortex and of co-activation of somatosensory areas. The ‘photic sneeze reflex’ is therefore not a classical reflex that occurs only at a brainstem or spinal cord level but, in stark contrast to many theories, involves also specific cortical areas.

## Introduction

Sneezing is most often induced by contact with infectious agents or inhalation of irritant dusts and chemical fumes [1], [2]. An unusual phenomenon known as the *photic sneeze reflex*, *sun sneeze* or *ACHOO (Autosomal Cholinergic Helio-Ophtalmologic Outburst) syndrome* has been described in the literature but has rarely undergone scientific investigation. This reflex is characterized by the induction of a sneeze upon sudden exposure of a dark-adapted subject to intensive bright light [3]. Usually, photic sneezing is evoked by sunlight, but Sedan [4] argues that artificial light, such as the light of an ophthalmoscope, photographic flash, or ultraviolet light, should also cause a sneeze. The photic sneeze reflex is clearly in need of study, especially in view of its high prevalence. A Swedish blood donor study [5] investigated one of the largest representative samples of subjects and reported photic sneezing in about 24% of the examined subjects. Photic sneezing was also demonstrated as occurring in babies, suggesting that some kind of congenital factors might influence this phenomenon [6]–[7]. Collie et al. [3] observed that the prevalence is higher in subjects with a family history of photic sneezing, and they therefore suggest an autosomal dominant inheritance, which they called the “ACHOO” (**A**utosomal **C**holinergic **H**elio-**O**phtalmologic **O**utburst) syndrome. However, the reported influence of inheritance on the photic sneeze reflex might be biased by an increased sensitivity among those who identified themselves as photic sneezers to the perception of the photic sneeze reflex among their relatives.

Although generally considered harmless, it has been hypothesized that photic sneezing is at least in part a causal factor in conduction deafness, mediastinorrhexis and cerebral hemorrhage [3]. Lang & Howland [8] point out that photic sneezing could be dangerous for individuals in certain professions, such as baseball outfielders, high-wire acrobats, and airplane pilots, or in commonly experienced situations such as driving out of a tunnel [9], which can triple the risk of sneezing.

A sneeze-evoking centre has been identified in the medulla of cats [10], but such a centre has yet to be confirmed in humans [11]. Generally, the sneeze reflex has two phases: An initial spasmodic inspiratory phase followed by a nasal and oral expiratory phase (described in detail by [1], [2], [11]). It is concluded that the sneezing reflex might be modulated by voluntary cortical mechanisms. Furthermore, Songa & Cingi [2] reported that sneezing could result from central nervous system pathologies, such as epilepsy or psychogenic pathologies. However, the neuroanatomy, neurophysiology and aetiology of the phenomenon are still unclear.

The optical-trigeminal summation theory suggests one possible explanation in the form of a kind of crosstalk between the optic and trigeminal nerves at the level of the mesencephalon [1]. It is hypothesized that intense light stimulation of the optic nerves results in cross-activation of the efferent maxillary branch of the trigeminal nerve. A second theory called “parasympathetic generalization“, posits that adjacently located parasympathetic branches are co-activated [1]. Activation of one particular branch of the parasympathetic nervous system might activate other branches. Thus, the projection of light on the retina stimulates pupillary constriction and to some extent lacrimation responses. A sufficiently intensive stimulus could therefore cause neural generalization that might lead to nasal congestion and a subsequent “tickling sensation”. According to Brubacker [12], the “tickling” sensation can be produced by the reflex onset of nasal congestion and secretion, and this is neurally transmitted to the brain where the motor execution of a sneeze is initiated. Other cases of parasympathetic generalization are well described. For example, reading with unsuitable glasses affects not only the third nerve outflow but also gastric motility through vagal outflow, urination may be accompanied by moderate lacrimation, and emotional states can influence any or all levels of parasympathetic outflow [13]. A third theory focuses on the role of parasympathetic system as well [1], suggesting that photic sneezers may have a parasympathetic hypersensitivity particularly within the nasal mucosa.

As far as we know, no neuroscientific investigations have as yet sought to identify the neural correlates of photic sneezing, making it therefore difficult to evaluate the merits of these theoretical positions.

We designed the present study in order to examine the cortical underpinnings of photic sneezing. As photic sneezing is a fast reflex-like phenomenon we anticipated that fast cortical processes would be involved. We used therefore electroencephalographic (EEG) measures to exploit its unrivalled time-resolution. Using electrical tomographic measures, we planned to localize the intracerebral sources of EEG activity on a millisecond basis. We first hypothesized that photic sneezers show an enhanced neural reaction in striate and extrastriate brain areas to visual stimuli compared with control subjects. Second, based on results of Breitenbach et al. [14], we expected that a brighter stimulus would cause a stronger photic sneeze reaction mediated by a stronger neural activation in the visual areas.

## Methods

### Ethics Statement

This study was conducted according to the principles expressed in the Declaration of Helsinki. The study was approved by the Institutional Review Board of “Spezialisierte Unterkommision Psychiatrie” (E08/2006). All participants provided written informed consent for the collection of samples and subsequent analysis.

### Subjects

Ten photic sneezers were recruited for this study. They reported experience of sneezing or prickle in the nose while looking into the bright light of a lamp or at the sun. In order to evaluate their photic sneezing experiences, we interviewed the subjects with a standardized questionnaire about different aspects of photic sneezing (rigidity, frequency, strength, daytime, season, refractory period, stimuli, family incidence). They reported occurrence of photic sneezing in 43.1–70.7% of the cases when looking at errhine stimuli. Most subjects reported the occurrence of photic sneezes as mainly in the summer season (one subject in autumn, two in winter). All of them reported having to sneeze upon direct exposure of the eyes to sunlight. Some of them also sneezed in response to bright artificial light. The photic sneezers (PS) were matched with ten control subjects (CON) according to age and sex (mean age ± standard deviation: PS 25.6±5.4, CON 26.1±4.6; groups did not differ significantly in terms of age: p = 0.65). All participants were undergraduate, healthy, consistent right-handed students, except for one left-handed subject in each group, according to the Annett-Handedness-Questionnaire [15]. Each group comprised 5 women and 5 men.

### Experimental Setup

Photic sneezers were compared with control subjects in terms of their neural responses to visual stimuli. Two experimental blocks were implemented (block 1 and block 2). In block 1, general differences in the visual system between the two groups were tested with a standardized checkerboard-paradigm. In block 2, neural correlates of photic sneezing were investigated by evoking this reflex by presentation of flashing lights. A break of five minutes was inserted between the two blocks.

### Block 1: Comparing Photic Sneezers with Control Subjects

In the first experimental block, we used a standard visual paradigm to stimulate the visual system [16]–[17]. This paradigm involved the presentation of a checkerboard of 16×16 black and white fields and a red fixation dot located in the middle of the visual field. Every 400 ms, black fields switched to white and white to black. The red dot was presented continuously. The presentation lasted 60 sec. Subjects were told to fixate the red dot. The scenario was presented on a computer screen (diagonal screen size  = 43.18 cm covering a visual angle of 34.3° at 70 cm from the subject's eyes).

### Block 2: Comparing Cortical Activations during Strong vs. Weak Prickle Sensations

In the second experimental block, light flashes were presented only to the photic sneezers. For stimulus presentation, a NEC video projector (model VT560 Lamptype: VT60LP; 50/50 Hz 2.9/1.4 A) projected a black fixation cross onto a reflective aluminium board (104 cm×80 cm). The board was positioned 230 cm in front of the subject's eyes (25.5° visual angle). The experiment comprised of three types of trials presented in randomized sequence, each trial type having a different degree of brightness. In each trial, a flash of constant brightness with a duration of 250 ms was presented 200 times. The interstimulus-interval (ISI) was randomly varied between 1000 ms and 2000 ms. Thus, one trial lasted about 6.25 min. The brightness of the flash was defined as ‘white’ ( = 78 Lum), ‘light gray’ ( = 16.5 Lum), ‘dark gray’ ( = 7.07 Lum). The subjects were instructed to focus the fixation cross in the centre of the aluminium board. After every trial, photic sneezers were asked three questions about their subjective experience of sneezing (occurrence, frequency and changes of the “tickling” sensation). The rating of the subjective intensity of the sneezing sensation was used for further analysis.

### EEG Recordings and Pre-Processing

For EEG measurement a 30-channel EEG system with the 10–10 system was used (Fp1/2, F3/4, F7/8, Fz, FT7/8, FC3/4, FCz, T7/8, C3/4, Cz, TP7/8, CP3/4, CPz, P7/8, P3/4, Pz, O1/2, Oz; BrainAmp system of BrainProducts, Munich, Germany). In addition, two EOG channels were co-recorded, located below the left and right outer canthi of the eyes. The recording reference was at FCz, with off-line re-referencing to the average reference. Digital sampling rate was 500 Hz, on-line filtering of 0.1–100 Hz, off-line bandpass filtering from 0.5 to 30 Hz, notch filtering at 50 Hz, impedance was kept below 10 kOhm. Subjects sat comfortably in a chair while viewing the stimuli. A head mount minimized movements and muscle artefacts.

### Global Field Power Analysis

After recording, each EEG sweep was visually inspected and trials with sweating artefacts, eye blinks, or eye movements excluded. Thus, ERPs (event-related potentials) were computed for each condition and subject on the basis of artefact-free EEG sweeps. ERPs of the groups were statistically compared by analyzing the Global Field Power (GFP). GFP constitutes a single, reference-independent measure of response strength. GFP was first introduced by Lehmann and Skrandies [18]. Mathematically, GFP is equivalent to the standard deviation of the potentials across all electrodes.

For ERPs, the resultant GFP waveform is a measure of potential (µV) as a function of time. GFP is a marker of the strength of a recorded scalp potential field [19]. By calculating the GFP for each subject and condition, changes in electric field strength can be identified. In this study, GFP were assessed statistically by comparing the GFP between conditions and between groups time point by time point. A false discovery rate (FDR) correction was applied to correct for multiple testing [20; 21]. FDR corrects for multiple time-point testing. It is a method for controlling accidental rejections of the H_0_ hypothesis when testing large datasets (threshold was set at p<0.05) [21], [22]

For statistical analysis of block 1, we first performed a between-groups test (PS vs. CON) using t-tests for independent samples at each time point (timeframes). The statistical significance of these tests was evaluated using FDR correction. We only report significant differences surviving this strict and conservative statistical thresholding.

**Figure 1:**
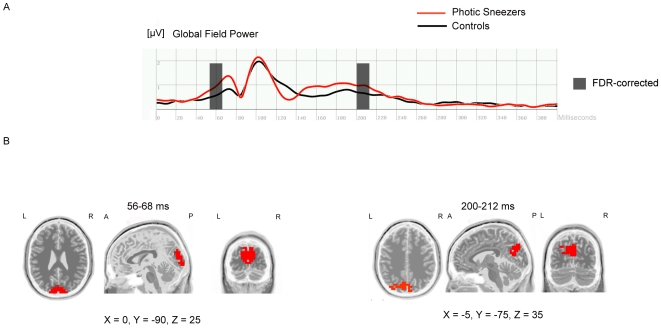
Comparing photic sneezers with control subjects. (A) Visual event-related potential (global field power) 0–400 ms after stimulus onset of block1 (checkerboard-paradigm) for both groups (photic sneezers vs. controls). Two time segments at 56–68 ms and 200–212 ms after stimulus presentation survived the FDR-correction (p<0.05). These time segments are marked by transparent rectangles. (B) sLORETA-analysis of the FDR-corrected time segments revealed significantly increased activity of the photic sneezers compared with control subjects. Neural generators for the time segment 56–68 ms are located in the primary visual cortex. The increased activation for the time segment 200–212 ms was found in the secondary visual cortex. Cortical activation differences estimated with sLORETA are displayed in red. X, Y, Z MNI-coordinates of the local maximum of the activation difference.

**Figure 2:**
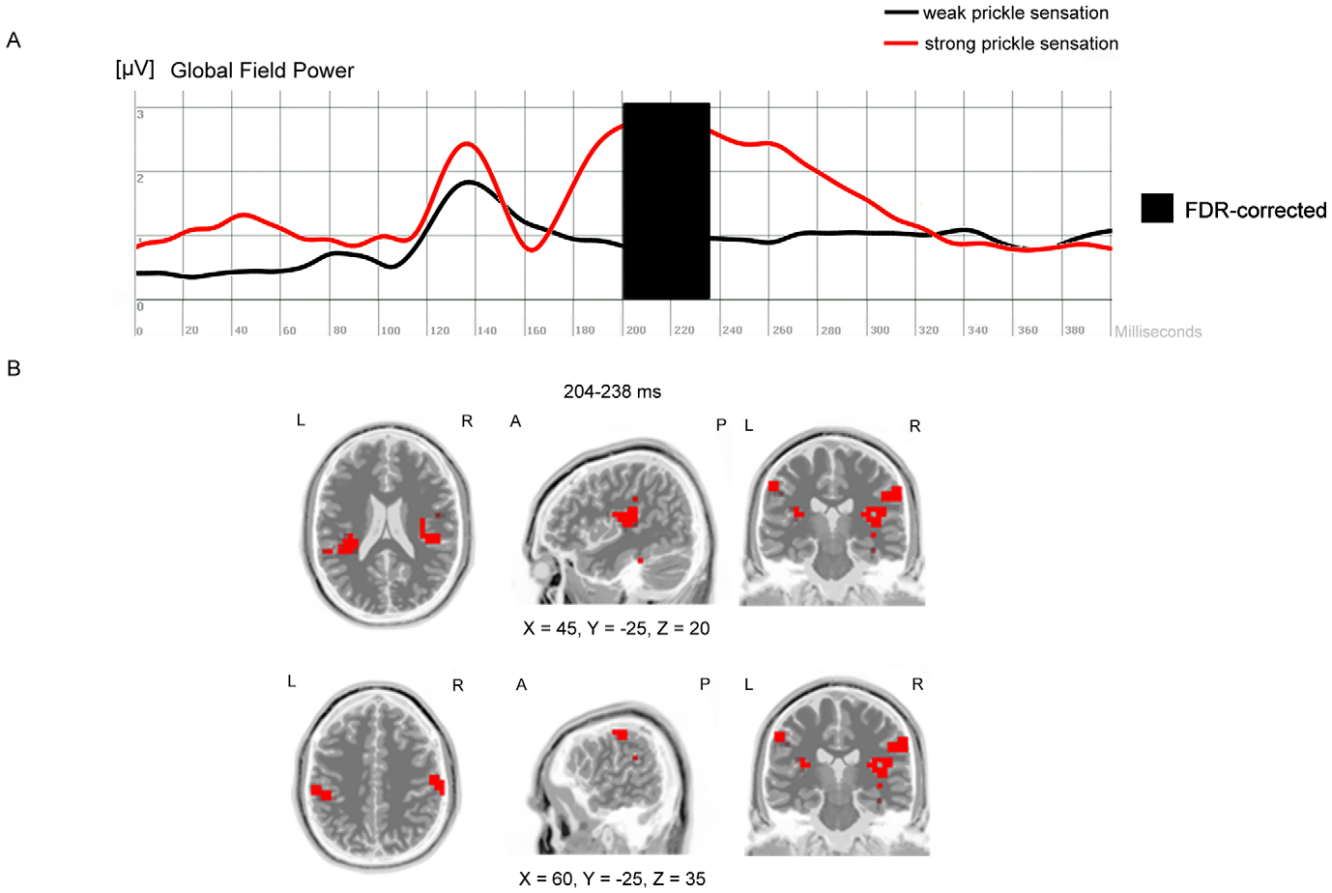
Comparing cortical activations during strong vs. weak prickle sensations. (A) Visual event-related potential (global field power) 0–400 ms after stimulus onset of block2 (flash-presentation) for both conditions (subjectively strong vs. weak prickle sensation) within the group of photic sneezers. FDR corrected significant differences in global field power were found at 204–238 ms after stimulus presentation. (B) sLORETA-analysis for this time segment revealed significantly (p<0.05) enhanced activity in the insula and secondary somatosensory cortex in the “strong prickle” condition. Cortical activation differences estimated with sLORETA are displayed in red. X, Y, Z MNI-coordinates of the local maximum of the activation difference.

For the analysis of block 2, the rating of the subjective intensity of the sneezing sensation was used and related to the cortical activation measures. This analysis therefore includes only the group of photic sneezers (PS). We identified the trials eliciting the subjectively strongest tickling sensations and compared them with those trials evoking the subjectively weakest tickling sensations (PSstrong vs. PSweak). For these trials the GFPs were compared using a t test for dependent samples separately for each time point. As for block 1, we only report the FDR-corrected results. In order to visualize the significantly different GFP time points surviving our conservative statistical threshold these are marked by transparent rectangles overlaid onto the corresponding GFPs in Figs. 1 and 2.

### Low Resolution Brain Electromagnetic Tomography (sLORETA)

In a second analysis, sLORETA (standardized low resolution brain electromagnetic tomography) software (publicly available free academic software at [23]) was used to localize the intracerebral dipoles of the scalp-recorded electrical potentials [24]. sLORETA is a method that computes a three dimensional distribution of electrically active dipoles (neuronal generator) in the brain as a current density value (A/m^2^) based on the recorded scalp electric potential differences [24]. sLORETA reveals an estimated solution of the inverse problem based on the assumption that the smoothest of all possible activities is the most plausible one. This assumption is supported by neurophysiological data demonstrating that neighbouring neuronal populations show highly correlated activity [24; 25; 26]. The sLORETA version used here is a standardized version of the minimum norm solution implemented in the frequently used older version of LORETA [24; 26]. Due to the low spatial resolution property of sLORETA, it should be kept in mind that localization results might suffer from some uncertainty in spatial extent. A three-shell spherical head model and EEG electrode coordinates derived from cross-registrations between spherical and realistic head geometry is utilized, both registered to the digitized MRI available at the *Brain Imaging Centre, Montreal Neurologic Institute* [27]. Computations are performed on a regular cubic grid at 5 mm resolution, producing a total of 6392 cortical grey matter voxels. sLORETA provides an estimation of the solution of the inverse problem by taking into account the well-known effects of the head as a volume conductor. Conventional LORETA and modern sLORETA analyses have been frequently used in previous experiments to localize brain activations on the basis of EEG or MEG data [28; 29; 30].

For the statistical analysis of sLORETA data (current densities) we are relying on the time segments we have identified with the procedures mentioned above. Differences (between groups or conditions) in the activity of the estimated intracerebral sources are determined on the basis of voxel-by-voxel t-tests of the current density magnitude. Statistical significance is assessed by means of a nonparametric randomization test [31], correcting for multiple comparisons. For the ERP data obtained in block 1, the computed current density magnitudes were statistically compared between both groups. For the estimated current densities obtained in block2 the inverse solutions were compared between both conditions (PS strong vs. PS weak). The statistical thresholds were set to a p<0.05 (corrected for multiple comparisons).

## Results

### Behavioural Data Analysis

We found that the trial with the brightest flashes did not consistently evoke the strongest prickling sensation in every subject's nose. Because we were interested in the source of the subjective sneezing sensation and not merely the effects of different degrees of brightness on brain activity, we decided to use the participants' subjective ratings of photic sneezing sensation for block 2 analyses.

### Comparing Photic Sneezers with Control Subjects

First, the Global Field Power (GFP) analysis in block 1 revealed two “time segments” during which the GFP between photic sneezers and controls differed significantly (FDR-corrected; p<0.05). The two time segments were found at 56–68 ms and at 200–212 ms after stimulus onset. The sLORETA procedure for locating the intracerebral sources of the electrical brain activations at these time segments revealed increased neural activation in the primary and secondary visual cortex of photic sneezers (Figure 1).

### Comparing Cortical Activations during Strong vs. Weak Prickle Sensations

For block2 the GFPs only from the “photic sneezers” were analyzed. The GFPs obtained during strong and weak prickle sensations were statistically compared separately for each time point. Thus, we used the subjective rating of prickling sensation associated with each trial. We identified for each subject the trials with the most strongest and weakest prickling sensations. These trials were used for statistical analysis. The GFPs obtained for the weak and strong prickling sensations were subjected to further statistical tests and revealed significant differences for the time segments 204–238 ms (p<0.05, FDR corrected for multiple comparisons). The subsequently performed sLORETA-analysis for the ERP data at this time segment identified significantly increased intracerebral activations in the insula and in the secondary somatosensory cortex (Figure 2).

## Discussion

The present study is the first systematic investigation of the photic sneeze phenomenon. We identified three main findings: (1) Photic sneezers generally demonstrated stronger intracerebral activations compared with controls within the primary and secondary visual cortex. This increased neural activation occurred at 56–68 ms and 200–212 ms after stimulus presentation onset. (2) The brightest flashes failed to consistently evoke the strongest prickling sensation in the photic sneezer's nose. (3) The subjective intensity of nose prickling was associated with a distinct intracerebral activation pattern such that the visual stimuli that evoked the strongest prickling sensation were associated with the strongest intracerebral activations in the insula and secondary somatosensory cortex.

The present findings can be interpreted in the context of increased attention, anticipation, and enhanced processing by photic sneezers of visual stimuli that evoke prickling sensations. Increased attention to and anticipation of specific (i.e., salient) stimuli are associated with increased activations in the secondary perceptual areas and in areas involved in emotional and cognitive processing of these stimuli [32; 33; 34; 35].

The finding of different cortical activations at approximately 200 ms after stimulus onset is in close correspondence with the findings and various interpretations associated with the P2 component evoked in classical ERP experiments. An increased P2 amplitude (especially at Pz) after presentation of invalid, emotional or salient stimuli has frequently been reported (e.g., [36; 37]). In this context, our results might be understood as indicating that the prickle-evoking visual stimuli (associated with unpleasant sensations in the nose) evoke enhanced stimulus processing in response to the specific salience of these stimuli for our subjects. The insula activation we identified during the prickling sensation corresponds closely with the insula activation found in several brain imaging studies during the presentation of unpleasant stimuli (e.g. pain [38], or disgust [39; 40]).

In addition, several functional imaging studies indicate the role of the insula in processing the link between bodily actions and sensations with emotional experience [41]. The insula is reciprocally connected with the secondary somatosensory cortex (S2) [42; 43; 44], this pointing to the crucial role of the insula in processing body representations in the context of emotional reactions. One could also speculate that in photic sneezers the visual stimuli activate the somatosensory pain-pathway, with the ascending thalamo-cortical somatosensory projections leading to enhanced activation of the insula and the secondary somatosensory cortex. In keeping with the aforementioned interplay between the insula and somatosensory cortex, we identified simultaneous activity between these regions.

Although anatomical localisations on the basis of EEG scalp measures using techniques like sLORETA should be interpreted with caution (due to the blurring nature of sLORETA and the relatively small number of 30 scalp electrodes), the identified brain regions are highly plausible. The maxima of the identified intracerebral sources during prickling sensation in photic sneezers are located in the lateral parts of the somatosensory cortex close to the somatotopic representation of the nose. This increase shows that the somatosensory area plays a crucial role in this phenomenon and supports the role of the cortex in the photic sneeze effect and mitigates the role of brainstem related reflexes. This assumption is also supported by the reports of many photic sneezers that the reflex can at least partially be suppressed voluntarily [45] implying cortical involvement. However, exposure of photic sneezers to bright light does induce visual overstimulation that can in turn cause a cascade of reactions that finally initiate a sneeze. Whether the enhanced activation in the primary and secondary visual cortices in response to visual stimuli could be explained by enhanced attentional processes is controversial. Noesselt et al. [33] demonstrated that the modulatory impact of attention on primary and secondary visual cortices cannot be identified before 140–250 ms after stimulus presentation, and that the primary visual cortex is modulated by “re-entrant” attentional mechanisms. In contrast to this study, the work by Pourtois et al. [46] and Stolarova et al. [47] suggest an early modulation of primary visual cortex by attention, emotion and learning. It is therefore unclear why early responses in the primary visual cortex are different in photic sneezers.

In analogy to synaesthetes, one might assume that photic sneezers show a different kind of neural organisation of the visual cortex in addition to an increased ocular sensitivity to light [48]. This specific organisation may be the result of altered development during brain maturation. For example, Buckley [49] observed an apparent higher prevalence of the photic sneeze reflex in children that subsides during adulthood. But there is at present insufficient data to assess this suggestion. A further possibility is that photic sneezers anticipate exposure to visual stimuli differently than normal control subjects: It is conceivable that they show a tonic increase in the activation level within the primary and secondary visual cortex in anticipation of those visual stimuli evoking unpleasant nose prickling sensations.

In summary, our results demonstrate that (1) photic sneezers have, as hypothesized, a generally enhanced excitability of visual cortex to standard visual stimuli, (2) a stronger prickle sensation in the nose of photic sneezers was associated with both activation in the insula and (3) stronger activation in the secondary somatosensory cortex.

We propose that the activation pattern of the somatosensory area is associated with overstimulation in the visual cortex in response to visual stimulation. To better understand the precise role of subcortical areas in photic sneezing (as proposed by Everett [1]), additional experiments are needed using methods that permit investigation of anatomical and functional differences in subcortical areas (such as high-resolution magnetic resonance tomography).

Thus, the results of this study do not contradict those theories [1] that emphasize the role of reflex pathway in the brain stem of photic sneezers. The present results do however support the view that even cortical circuits rather than brainstem circuits might play a pivotal role in controlling (or modulating) this extraordinary and rarely investigated behaviour.
